# Single cell analysis to dissect molecular heterogeneity and disease evolution in metastatic melanoma

**DOI:** 10.1038/s41419-019-2048-5

**Published:** 2019-10-31

**Authors:** Luigi Fattore, Ciro Francesco Ruggiero, Domenico Liguoro, Rita Mancini, Gennaro Ciliberto

**Affiliations:** 1 Department of Molecular and Clinical Medicine, Sapienza University of Rome, Laboratory affiliated to Istituto Pasteur Italia-Fondazione Cenci Bolognetti, Rome, Italy; 2 IRCCS, Regina Elena National Cancer Institute, Rome, Italy; 3 Department of Molecular and Clinical Medicine, Sapienza University of Rome, Rome, Italy

**Keywords:** Melanoma, Cell biology

## Abstract

Originally described as interpatient variability, tumour heterogeneity has now been demonstrated to occur intrapatiently, within the same lesion, or in different lesions of the same patient. Tumour heterogeneity involves both genetic and epigenetic changes. Intrapatient heterogeneity is responsible for generating subpopulations of cancer cells which undergo clonal evolution with time. Tumour heterogeneity develops also as a consequence of the selective pressure imposed by the immune system. It has been demonstrated that tumour heterogeneity and different spatiotemporal interactions between all the cellular compontents within the tumour microenvironment lead to cancer adaptation and to therapeutic pressure. In this context, the recent advent of single cell analysis approaches which are able to better study tumour heterogeneity from the genomic, transcriptomic and proteomic standpoint represent a major technological breakthrough. In this review, using metastatic melanoma as a prototypical example, we will focus on applying single cell analyses to the study of clonal trajectories which guide the evolution of drug resistance to targeted therapy.

## Facts

- Intratumoural heterogeneity is a major obstacle for the clinical efficacy of anticancer drugs as in the case of targeted/immuno-therapy in metastatic melanoma
- Single cell approaches directed towards studying the individual cellular elements of the tumour and its microenvironment are formidable tools for uncovering the driving forces of heterogeneity from the genomic, transcriptomic and proteomic perspectives
- Ab initio drug resistant transcriptional programs are present before starting targeted/immuno-therapies and guide development of resistance.

## Open questions

- Do different differentiative vs. invasive cellular states coexist in “preset” conditions? Or are they “interconvertible” and follow drug treatment or immunologiocal pressure where one of the two emerges over the other?
- Which are the molecular basis of T cell residency as a determinant of ICIs failure/response focusing on a single cell level?
- Can non invasive liquid biopsies help implement the power of single cell approaches for diagnostic purposes?

## Introduction

**Fig. 1:**
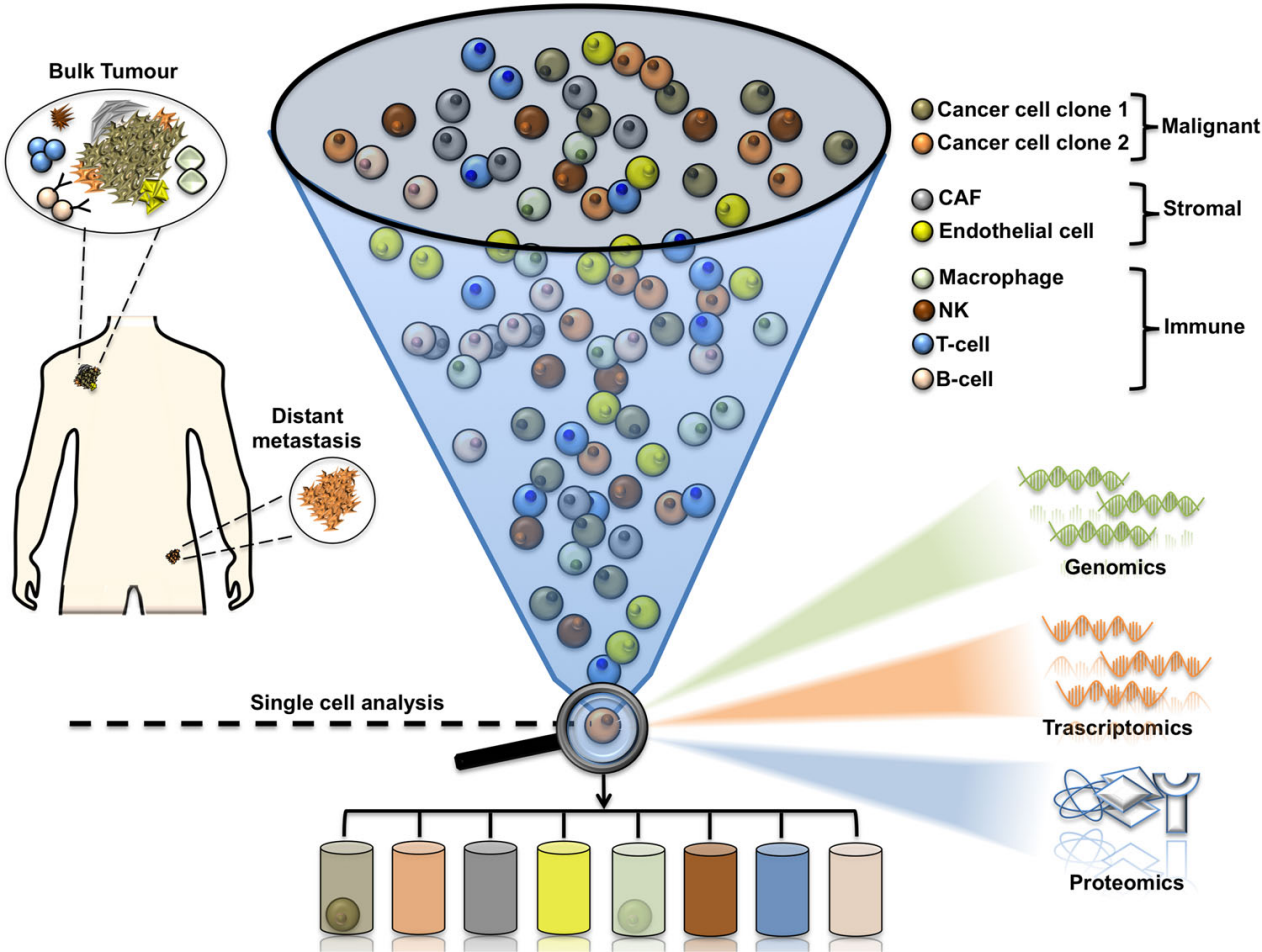
Schematic diagram illustrating single cell analysis ability to solve intratumor heterogeneity. Bulk tumour is constituted by different cellular elements of malignant, stromal and immune origins whose molecular state is difficult to determine when considered all together. Furthermore, bulk tumours can also contain malignant cells with different trascriptomic programs which help them to metastatize or resist antineoplastic agents. Single cell approaches are emerging as valuable tools in dissecting those complexities from genomic, transcriptomic and proteomic perspectives and in potentially determining the molecular signatures of every cell and its destiny during the course of the disease

The transformation of malignant cells is a process which encompasses the acquisition of sequential alterations that however do not occur syncronously within the initial growing tumour mass. Thereby, cancers generally become heterogeneous during the course of the disease^1,2^. This heterogeneity is driven by genetic, transcriptomic, epigenetic, and/or phenotypic changes which result in different levels of sensitivity to antineoplastic therapies^3^. In cancer biology, this feature can be roughly differentiated into interpatient and intratumour/intrapatient heterogeneity^1^. The first one has long been recognized, since tumours of the same histological type belonging to different patients do not share the same biological features and clinical evolution^4^. Differently, intratumor heterogeneity is characterized by the existence of distinct cellular populations within tumours^4^ and can manifest as spatial or temporal variations^1^ (Box 1). Among the influencers of tumour heterogeneity an undisputed role is played by the pressure imposed from host immune system^4,5^. Indeed, immunosurveillance favours the emergence of subclonal populations characterized by the lack of immunogenic antigen expression hidden from immune attack (immunoediting)^6,7^. Thereby, cancer cells induce the development of an immune-suppressive microenvironment characterized by both altered cellular and non cellular elements^4,7^. The first ones are represented by tumour-associated macrophages (TAMs), cancer-associated fibroblasts (CAFs), T cells and myeloid-derived suppressor cells (MDSCs), whereas examples of the latter are programmed cell death ligand 1 (PD-L1) and anti-inflammatory cytokines like TGF-β (transforming growth factor beta)^4,8^. Given the great complexity of intratumor heterogeneity, it is clear that bulk tumours’ study in its totality is insufficient. Hence, the recent advent of single cell (sc) analyses provides unique opportunities to dissect these complexities from genomic, transcriptomic and proteomic points of view (Fig. 1)^9–13^ and is emerging as a major technological breakthrough (Box 2). However, it is important to point out that large-scale sc proteomics are still hampered by several obstacles differently from acid nucleic-based protocols. Importantly, given the aforementioned huge impact of the tumour microenvironment in intratumour heterogeneity sc approaches can also serve to assess the malignant, microenvironmental, immunologic and metabolomic states that characterize tumorigenesis as well as the response to pharmacological pressures^14^. In this review, we have decided to focus on one of the most aggressive and heterogeneous cancers, i.e., metastatic melanoma (Box 3)^6,15,16^, which has been the focus of several sc applications over the last few years. In particular, we will assess the most relevant studies that aimed to unveil the clonal trajectories which guide the development of this tumour and especially the establishment of resistance to targeted/immuno-therapies.

Box 1 Spatial and temporal heterogeneity

- Spatial heterogeneity is marked up by the uneven distribution of genetically and/or epigenetically different tumour subpopulations present in the same tumour lesions or in syncronous lesions within the same patients^1^.
- Temporal heterogeneity refers to dynamic variations of tumour cells over time and can be originated, for example, in response to the pressure of antineoplastic treatments which concur to induce further genetic instability in tumour cells and to counteract innate and/or adaptive immunity^1^.

Box 2 Technological breakthroughs of single cell approaches

- Single cell genomics. Arising from genome sequencing, it requires whole-genome amplification in order to gain sufficient material for sequencing since DNA yielding from single cell samples is usually limited. Gene amplification bias limits the efficacy of this approach and causes suboptimal genome coverage. This issue has been technically solved through different approaches such as (1) multiple displacement amplification (MDA) and (2) looping-based amplification cycles (MALBAC)^9^. The first one is based on the annealing of random hexamers to denatured DNA followed by isothermal strand-displacement synthesis of genome products whereas the latter ensures that DNA products cannot be used as template for undesired amplification through the achievement of quasilinear amplification.
- Single cell trascriptomics. It is the most frequently used approach and is based on single-cell RNA sequencing (scRNA-seq)^10^. Common steps are: (1) cell lysis; (2) reverse transcription to obtain first strand cDNA; (3) synthesis of the second strand and (4) further amplification of the products. Recently, the advent of single-cell bar-coding added to mRNAs during reverse transcription has reduced the limitations related to the biases due to PCR amplification cycles^8^. This method is usually implemented by a multiplex sequencing in order to obtain a numbers of libraries which are pooled and sequenced simultaneously with a single run.
- Single cell proteomics. Differently from acid nucleic-based approaches full-scale proteomic analyses are yet to follow at single cell level. Flow cytometry is historically the most widely used and can be also implemented with cell sorting isolations (FACS) through the use of fluorophore-conjugated antibodies^9^. The evolution of this technique is mass cytometry by CyTOF^9^ (cytometry by time of flight) which uses antibodies labeled with metal isotopes without significant overlap thus not requiring any compensation as in the case of fluorescence for FACS analysis. Moreover, it allows the detection of more than 40 unique markers as compared to the 8–12 parameters comprised in a canonical flow cytometry panel. Another approach is the microfluidics-based single-cell barcode chip (SCBC)^39^ technology which takes advantages of panels containing several microchambers engineered for cell lysis and protein capture with appropriate antibodies through a sandwich immunofluorescence-based assay.
- Digital Spatial Profiling. This novel platform developed by NanoString allows a high throughput detection of protein and/or RNA at single cell level from single formalin fixed paraffin-embedded (FFPE) sample tissue section (up to 96 proteins and over 1000 RNA targets). Briefly, the assay relies on antibody and/or RNA probes coupled to oligonucleotide tags, which are able to bind different regions of the same section according to the expression of specific markers. Hereafter, the oligonucleotide tags are decoupled from the target via UV exposure and then they are quantitated in a NanoString nCounter assay. Results allow to map the tags/targets back to tissue location, yielding a spatial overview of intratumour heterogeneity in the tissue section tested.

Box 3 Main mutations of human melanoma based on TCGA data According to The Cancer Genome Atlas (TCGA)12 mutation frequencies in melanoma range across 0.1–100/Mb with a mean of 16.8 mutations/Mb. Based on the most prevalent mutated genes the genomic classification of this tumour foresees four subtypes: mutant BRAF, mutant RAS, mutant NF1, and Triple-WT (wild-type). More specifically, 50% of all melanomas harbour BRAF (v-Raf murine sarcoma viral oncogene homolog B) V600 mutations, 15–30% NRAS (neuroblastoma RAS viral oncogene homolog) mutations, whereas NF-1 gene is altered in 12–18% of cases. Hence, the mitogen-activated protein kinase (MAPK) pathway is the most relevant oncogenic signaling altered in melanoma, an evidence that guided the development of targeted therapies using BRAF and MEK inhibitors.

## Single cell profiles to address melanoma development and progression

The first study that aimed to explore in depth the distinct genotypic and phenotypic states of melanoma at sc level was performed by Tirosh et al. in 2016^17^. These authors evaluated through single cell-RNAseq (sc-RNAseq) the profiles of 4645 cells represented by immune, malignant and stromal cells isolated from different melanoma patients and divided through sorting into CD45+ and CD45–, respectively. Hereafter, cell cycle phase–specific signatures distinguished cancer cells into cycling and non-cycling cells. The first ones were characterized by genes like cyclin D3 whereas, in contrast, the histone demethylase JARID1B was associated with non-cycling cells^17–19^. Hereafter, spatial intratumour heterogeneity was assessed through sc-RNAseq of malignant cells derived from four distinct regions of the same treatment-naïve tumour following surgical resection. Of note, this type of study was impossible to be pursued using canonical bulk RNA-seq. Interestingly, malignant cells originating from one of these regions were characterized by a peculiar trascriptomic signature composed of several oncogenes, such as FOS, JUN and NFκB. The same approach was used to dissect the tumour ecosystem in response to treatments with MAPK inhibitors and immune checkpoint blockade: two aspects which will be discussed in the next section.

Also Gerber et al. took advantage of sc-RNAseq to study melanoma cells deriving from three different patients with BRAF^wt^/NRAS^wt^, BRAF^mut^/NRAS^wt^ and BRAF^wt^/NRAS^mut^ and grown in vitro at low passages^20^. Cell subpopulations were clustered into three major groups: (1) proliferative, (2) pigmented and (3) stromal. Interestingly, most cells were characterized by genes involved in cellular proliferation, DNA replication/repair and mitosis (group 1). Group 2 of pigmented cells was characterized by genes associated with the master regulator of melanocyte “MITF^high^”, whereas stromal group showed receptor tyrosine kinase “AXL^high^” expression levels. Furthermore, these authors sought to identify peculiar signatures of genes capable of separating the three different types of melanomas. Specifically, BRAF^wt^/NRAS^wt^ cells were characterized by mixed oxphos/pigmentation signatures and by specific stromal cell genes that were upregulated only in this subset. Differently from the others, the transcriptomic alterations of BRAF^mut^/NRAS^wt^ cells were found to be governed by large-scale copy number variations^20,21^ and were enriched in genes like CD36, CBR1 and SNX10. Finally, NRAS^mut^ cells mostly overexpressed genes associated with the stromal signature. It should be noted that, a significant fraction of cells belonging to all the three low passage cultures shared a common signature of proliferative genes. Differently, cells expressing JARID1B constituted a slow proliferative population and, coherently with their stem-like features, were only a small percentage^22^ of the total. Finally, regarding the proposed antagonism of MITF/AXL-related transcriptional programs it was shown that BRAF^mut^ cells mostly activated MITF induced genes wheras in contrast NRAS^mut^ cells were enriched in the AXL program. Unfailingly, double wild-type tumours revealed mixed MITF/AXL characteristics.

In addition, Wirth et al. analyzed three short-term melanoma cultures representing the same aforementioned genetic subtypes through sc-RNAseq^23^. They opted to study temporal intratumour heterogeneity through pseudotime (PT) dynamics of the cell population to track gene regulatory programs during cancer progression. Again, cells were assigned to proliferative, stromal and/or pigmented groups. Genes correlated with proliferation described PT progression in all the three melanoma subtypes. Differently, gene stromal signatures involved in extracellular matrix interaction with cancer cells showed to peak early during cancer progression. Pigmentation signatures presented a high prevalence in the double wild-type cells, low activity in NRAS^mut^ and fluctuating expression in BRAF^mut^. It is important to note that the differentiative/pigmented state correlated with MITF, MLANA and S100 calcium-binding protein B^24^ expression in opposition to AXL and nerve growth factor receptor (NGFR), respectively.

Recently Kunz et al. also performed a comprehensive sc-RNA-seq analysis of different melanocytic nevi and primary melanomas in order to dissect the molecular mechanisms of melanomagenesis^25^. PT dynamics allowed to identify two distinct evolutionary transcriptomic trajectories for non malignant lesions and primary melanomas called type 1 and type 2, respectively. In particular, type 1 rewires melanoma specific differentiation genes such as again MLANA and MITF whereas, on the contrary, type 2 is associated with AXL expression together with CAFs, T cell-specific and inflammatory signatures. From a prognostic perspective, in regards to the MAPKi resistance signature, the first type showed an enrichment of BRAF, cMET and YAP1 oncogenes whereas type 2 was characterized by the so-called IPRES signature of innate resistance to anti-PD-1 immunotherapy previously identified^26^.

The thread linking all these studies is the existence of two main opposite phenotypes involving AXL vs. MITF signatures; a paradigm which gains further interest in the context of resistance to targeted therapy^27–30^ (see the section “Targeted therapy”).

Finally, a totally different study by Kumar et al. took advantage of different treatment-naive syngeneic mouse models including melanoma to address via sc-RNAseq cell-cell comunications within the tumour microenvironment in order to understand how non malignant cells cooperate with cancer cells to facilitate tumour growth and dissemination^31^. Thanks to this approach, it was demonstrated that both CAFs and endothelial cells are the main cellular determinants of these processes from different points of view. On one hand, they up-regulate collagens which bind to either CD93 and/or integrin receptors on tumour cells to positively induce tumour growth. On the other hand, they produce proteins like metallopeptidases (MMPs) and metalloproteinases (TIMPs) to allow cancer cell metastatization from the primary tumour site. Table 1 summarizes the studies described in this paragraph.

**Table 1:** Single cell studies in melanoma development and progression

Authors	Samples	Approach	Main Markers and States
Tirosh et al.^17^	4645 cells (malignant, immune, and stromal cells) from 19 melanoma patients	-Sorting/FACS -sc-RNAseq	-JARID1B (slow cycling melanoma cells) -ATF3, FOS, FOSB, JUN, JUNB (malignancy state) -EGR1/2/3, NDRG, HSPA1B (stress response) -NF-kB (resistance to MAPKi) -MITF/AXL (sensitivity/resistance to MAPKi)
Gerber et al.^20^	92 melanoma cells from 3 short-term cultures by three patients: -BRAF^wt^/NRAS^wt^ -BRAF^mut^/NRAS^wt^ -BRAF^wt^/NRAS^mut^	sc-RNAseq	-TOP2A, ASF1B, RRM2 (proliferative state) -MITF, PMEL, TRPM1, TYRP1 (oxphos/pigmented state) -AXL, VTN, POTEI, A2M (stromal state) -JARID1B (slow cycling melanoma cells)
Wirth et al.^23^	3 melanoma short-term cultures: -BRAF^wt^/NRAS^wt^ -BRAF^mut^/NRAS^wt^ -BRAF^wt^/NRAS^mut^	sc-RNAseq	-ANXA1/2, FN1, CALD1, SORBS2 (extracellular matrix remodelling) -MITF, CDH1, PMEL, TYR (differentiation) -AXL, NGFR (invasion, MAPKi resistance)
Kunz et al.^25^	-23 melanocytic nevi -57 primary melanomas	sc-RNAseq	-MITF, MLANA, TYR, MLPH (differentiation) -AXL, JUN, FOS (invasion)
Kumar et al.^31^	6 syngeneic mouse tumour models (>10,000 of malignant, stromal and immune cells). Melanoma, breast mammary carcinoma, lung carcinoma, 2 colon carcinomas, fibrosarcoma	sc-RNAseq	-CCR1, CCR2, CCR5, CCL2, CCL4, CCL12 (receptor-ligand interaction) -CD93 (tumour growth) -MMPs, TIMPs, ADAMs (invasion) -PD-L1/PD-1, CTLA4-CD80 and CTLA4-CD86 (immunosuppressive responses)

## Single cell analysis for studying resistance to therapy in melanoma

Targeted therapy and immunotherapy have revolutionized the fight against metastatic melanoma providing unprecedented benefits in terms of objective responses and overall survival^32^. However, this positive scenario is mitigated by the occurrence of drug resistance^33–35^. Among the genomic^36^ and non genomic^37–40^ mechanisms of resistance an undisputed role is played by intratumour heterogeneity. In this context, single-cell approaches are emerging as informative platforms having the potential to decipher the complex clonal relationships and to unravel the driving forces behind intratumoural heterogeneity in the context of resistance to MAPKi and ICI therapy in melanoma. This is the focus of the following part of this review.

### Targeted therapy

The pioneering study which analyzed at sc-level intratumor heterogeneity in the context of MAPKi resistance in melanoma is the aforementioned work by Tirosh et al^17^. The fundamental discovery of this study is to have demonstrated that, although the bulk tumour of each melanoma could be potentially cataloged as “MITF^high^” or “AXL^high^”, when this is analyzed at a single-cell level every tumour contains cancer cells which correspond to both transcriptional states. This conclusion stems from the identification of a dormant drug resistant “AXL^high^” subpopulation of cells in the treatment of naïve melanomas mostly characterized by “MITF^high^” programs. This small subpopulation of cells would have been otherwise undetectable through a classical bulk analysis of the tumour mass. Starting from the hypothesis that MAPKi treatment could facilitate the emergence of “AXL^high^” cells, RNA-seq analyses were performed on differently matched BRAF-mutant melanomas before and after resistance to MAPKi. Results confirmed the transcriptional shift from the MITF state toward the “AXL^high^” program in drug resistant samples.

Also Ho and colleagues took advantage of the scRNA-seq to study resistance to targeted therapy in melanoma^41^. Specifically, the differential transcriptomic signatures from different BRAF-mutant melanoma cell lines rendered resistant to a BRAFi in vitro were determined. Thanks to this approach, Dopachrome Tautomerase gene (DCT) was identified as the most upregulated gene in BRAFi-resistant cells. Importantly, this marker has not been previously identified using canonical bulk RNA-seq technologies. Based on the assumption that a BRAFi-resistant state is present before starting MAPKi, “DCT^high^” melanoma cells were sorted in the initial drug-sensitive population. These cells showed a greatly reduced response to BRAFi. Furthermore, scRNA-seq data also helped to identify a transitional intermediate subpopulation constituted of cells “committed” to developing MAPKi resistance. These few cells upregulated known oncogenes as AXL, JUN and NRG1 which were previously identified in MAPKi resistance in melanoma^42^. Rare (<1%) AXL/NRG1 positive cells sorted from the parental population, were coherently characterized by a reduced sensitivity to BRAF inhibition as compared to the rest of the cells.

The existence of a dormant drug resistant subpopulation before starting MAPKi treatment naturally resembles the concept of “minimal residual disease” (MRD). This may be driven by a small subpopulation of drug-tolerant cells able to survive upon drug exposure while the rest of the tumour cells are rapidly destroyed.

This aspect has been tackled through the use of sc-RNAseq carried out by Rambow and colleagues^43^. The model adopted to study MRD was represented by BRAF-mutant melanoma PDX mouse models exposed to MAPKi. In particular, treatments induced tumour shrinkage (phase 1) reaching an almost complete impalpable size (phase 2), then continuous MAPKi exposure led to the development of resistance (phase 3), which indicates the presence of MRD in phase 2. Hence, scRNA-seq was performed from individual melanoma cells that were isolated at different times in order to investigate their trascriptional states. First of all, investigators observed that “phase 2 cells” were characterized by four distinct transcriptional states. The first one, characterized by “MITF^high^” activity expresses known markers of differentiation/pigmentation, and was enriched in the transition from phase 1 to phase 2. The second state was called “invasive” because associated with epithelial-to-mesenchymal transition (EMT) markers and “MITF^low^” levels. The percentage of these cells was reduced from untreated tumours to phases 1 and 2, suggesting that drug tolerance is not driven by a switch from proliferative to invasive behaviour. The third cluster was characterized by a de-differentiative state of neural crest stem cell (NCSC) markers, enriched in genes like NGFR. Similar to the previous “invasive” cluster, also this one was “MITF^low^”, but in a different way, its proportion increased during drug administration. The latter state exhibited intermediate MITF levels and high expression of genes associated with nutrient-deprived cells (“starved-like” melanoma cells-SMCs). Critically, these particular cells increase in phase 1 and, to a lesser extent, in phase 2. These data allowed to define a model of transcription dynamics during MAPKi exposure, for which selective pressure firstly draws melanoma cells to enter in a transition state passing from a proliferative behaviour to an SMC state. At this point the possible crossroad for cells is to move along a pigmented trajectory (MITF^high^) or a de-differentiative path (MITF^low^) of invasive NCSCs. Hereafter, these findings have been validated through bulk RNA-seq performed on matched tumour samples from “drug-naive” and “on MAPKi treatment” melanoma patients. Interestingly, authors observed that NCSC program was the most predominant drug-tolerant state during MAPKi treatment. Finally, scRNA-seq data were analyzed to assess the molecular basis of NCSC programme, as mainly responsible for this drug-tolerant state. In this way a massive involvement of the retinoid X receptor-g (RXRG) and of its target de-differentiative genes, such as GFRA2, NGFR and SOX10 has been demonstrated. These findings clearly suggest the possibility of pharmacologically hitting the NCSC subpopulation through the inhibition of RXRG in order to delay drug resistance emergence. Coherently, the use of an RXR antagonist in combination with MAPKi demonstrated the capability to reduce the growth of PDX models and to delay time-to-disease progression in mice.

Melanoma cell state transition associated with MAPKi resistance has also been the focus of the work by Su and colleagues^44^. These investigators tested different patient-derived BRAF-mutant melanoma cell lines treated with a BRAFi from 3 days to 3 weeks. FACS analysis using known markers, i.e., MART-1 and NGFR, allowed them to cluster cell responses according to the phenotypic plasticity in response to BRAF inhibition. In detail cells with “plastic trajectories” start from a “pigmented” state “MART1^high^/MITF^high^”, and then transit into a slow-cycling neural crest-like state “NGFR^high^”. Continuous BRAFi administration led to a drug resistant MART1/NGFR double negative state. Having identified this plastic melanoma cell model, SCBC technology allowed to identify the activation of p-ERK and p-NFκB p65, signalling as mediators of tolerance to BRAF inhibitors. To determine the effects of simultaneously targeting BRAF together with p-ERK and pNFκB, melanoma cells were treated with combinations of Vemurafenib and/or Trametinib and/or an inhibitor of NFκB p65 nuclear translocation. Results demonstrated that only triple combination treatments were able to keep melanoma cells in the MART-1 drug-sensitive state. These data indicate that the nuclear translocation of NFκB is a pivotal event in mediating adaptive transition toward the BRAFi-tolerant phenotypes.

Finally, Lun and colleagues designed a multi-omic approach to study the role of human kinome/phosphatome in melanoma drug resistance through mass-cytometry single-cell analysis^45^. To be more specific, these authors transfected hundreds of kinase/phosphatase constructs in human embryonic kidney HEK293T cells which were then treated or not with EGF. The CyTOF evaluation allowed to observe that the activation of MAPK signalling dominated over all the other oncogenic pathways. However, the most revelant finding encompassed the evidence that several cells showed activated p-ERK1/2 in the absence of EGF stimulation. The bioinformatics analysis identified the effectors characterized by the strongest relationship with MAPK signalling, such as MAP3K8, MOS and SRC, which were then further investigated to test their relationship with MAPKi resistance in melanoma. A375 cells were treated with either BRAF and/or MEK inhibitor following the transient transfection of these three proteins. Results showed that MAP3K8 and MOS overexpressing cells were resistant to BRAFi but not to MEKi, suggesting that the activity of this kinase bypasses this inhibition. Conversely, A375 overexpressing SRC were characterized by ERK activation in the presence of all the drug regimens tested, indicating MEK-independency in this context. All together the results of this impressive study identified novel kinases able to reactivate MAPK signalling in melanoma not only following the sole mutant BRAF inhibition, but also the BRAF-MEK hit.

### Immunotherapy

As anticipated above, the sc-RNAseq analysis is a valid approach in studying the heterogeneity of the tumour microenvironment: an aspect which aquires particular relevance to better understand the mechanisms of resistance to immunotherapy. Tirosh et al. looked at patterns of tumour-infiltrating lymphocytes (TILs)^17^, which are the main cellular determinants of immunotherapy success/failure. Those cells were divided into three main subsets, i.e., CD4+, CD8+ and Tregs based on the expression levels of defined surface markers. The exhaustion program of each cell type was determined through the expression of known inhibitory receptors such as PD1, TIM3 and CTLA4. In this way, a signature of 28 genes was found to be strongly upregulated in exhausted T cells of most melanomas and was used to assess immunotherapeutic response. Furthermore, the expression levels of two coinhibitory receptors, namely PD1 and TIM3, were validated through IF staining. This study paved the way to capitalise on the lessons learned from sc-RNAseq of immune cells for predicting response to immunotherapy in melanoma.

The goal of identifying biomarkers of response to immunotherapy is at the center of Krieg’s et al. study which made use of the CyTOF mass cytometry^46^. These authors analyzed PBMC samples derived from melanoma patients before and after initiating anti-PD1 therapy. Cells were stained with three separate cytometry panels for: (1) lymphocytes, (2) T cell function and (3) myeloid cells. Thanks to this approach, two important lessons emerged. Firstly, in responding patients it was demonstrated that after starting therapy higher frequencies of central memory T and NKT cells were in circulation together with a more activated T cell compartment characterized by IL-4, IFN-γ and IL-10 cytokine production. As to the second point, activated classical monocytes were identified as a prerequisite for a successful response to ICIs therapy. In particular, CD14 + CD16b-HLA-DRhi monocytes were found to be the most highly represented myeloid cells whose frequency is predictive of anti-PD1 treatment response with improved patient survival before starting therapy. This finding emerges as a novel parameter to guide clinical decisions.

Importantly, Nirschl et al have helped to uncover through scRNA-seq the mechanisms which sustain immune system’s surveillance escape in melanoma^47^. These authors demonstrated that IFN-γ signalling regulates this program in particular through the expression of a the SOC2 member of the family of cytokine-induced Jak-Stat regulators. This effector was found to be upregulated in dendritic cells (DCs) derived from melanoma metastasis where it hinders the adaptive anti-tumoral immunity and DC-based priming of T cells. From a prognostic point of view, IFN-γ/SOCS2 programs have been correlated with the worst melanoma patients’ survival probability^47^.

The study by Jerby-Arnon et al. deserves particular mention because it unravels cell-cell interactions in the tumour ecosystem which are critical in tilting the balance for ICIs therapy efficacy^48^. In this context, one of the main parameters associated with prolonged therapy response is the amount of T cell infiltration within the tumour, a condition named “hot/high” or “cold/low”. The determinants of these states however, are only partially known. To investigate this aspect, cold tumour programs were bioinformatically determined by combining the scRNA-seq from different melanomas and bulk RNA-seq data from of T cell and malignant cell signatures belonging to TCGA. In this way, the so called “exclusion program” was identified as a malignant cell signature composed of genes whose expression was associated with T cell exclusion and immune evasion. This program was able to predict intrinsic resistance to ICIs since it was found to be upregulated in melanoma patients who did not respond to anti-PD-1 blockade as compared to responders before starting therapy.

Along the same lines, Sade-Feldman et al. investigated the profile of several immune cells derived from melanoma patients treated with different ICI therapies through sc-RNAseq^49^. In this way, 11 major clusters were identified: among them the most represented were those related to T cells. Hence, investigators focused on the two main clusters which were enriched in genes: (1) related to memory/activation and (2) exhaustion. The first one was increased in responding lesions, whereas the second one in non responding patients. Interestingly, both clusters coexisted in all pre-therapy lesions but in different proportions. Once again, this finding confirms the existence of initial responding *vs*. non-responding programs which emerge over the others during the course of the therapy and decide on the clinical response. The sc-RNA-seq data were also exploited to identify individual CD8+ T cell markers associated with response to therapy. Among them, the top marker associated with responding lesions was the transcription factor TCF7, which is pivotal for T cell differentiation, self renewal and memory^50^. In contrast, CD8+ T cells deriving from non responder patients mostly upregulated two surface exhaustion markers, namely CD39 and TIM3. Finally, this work sought to address the epigenetic landscape of CD8+ T cells leading to either states of exhaustion and memory. In order to do this, these authors took advantage of T cell sorting for the aforementioned CD39/TIM3 markers followed by open chromatin analysis using ATAC-seq. These results showed differentially accessible DNA regions in positive and negative CD8+ T cells containing genes related to either exhaustion and memory, respectively. It should be noted that searching transcription factors mostly enriched in open chromatin, the authors found BATF motifs (an exhaustion marker) in CD39/TIM3 double positive cells, in contrast to TCF7 in negative cells.

Finally, the impact of memory T cells on ICI therapy responses in melanoma was also assessed by Gide et al. which used CyTOF mass cytometry to identify subpopulations of these cells associated with response to anti-PD-1 and/or anti-CTLA-4 antibodies^51^. To this purpose, a custom panel composed of different markers of T cell differentiation was used. In this way, a highly abundant T cell population, namely CD45RO + EOMES+, was identified in patients who responded to combined immunotherapy. EOMES is a master regulator of T cell function and long-term memory of cytotoxic T cells^52^. Furthermore, those cells also upregulated CD69, a marker of tumour tissue residency for both CD4+ and CD8+ T cell subpopulations. These CD45RO + EOMES+ cells were also enriched for the TBET factor, which, together with EOMES, acts as master regulator of the T effector memory^52^. Finally, these CD8/CD4 + EOMES + CD69 + CD45RO+ memory T cells were associated with longer PFS in response to anti-PD-1 therapies in melanoma patients. Of note, it will be of interest to test the predictive value of this signature in a cohort of melanoma patients who fail to respond to anti-PD-1 checkpoint blockade and then respond to combined immunotherapy or viceversa.

All together the studies described in these two sections demonstrate how sc approaches can provide an unprecedented view of intratumor heterogeneity impact on the development of resistance to targeted/immuno-therapies in melanoma.

Table 2 schematically summarizes the main findings of these studies.

**Table 2:** Single cell studies in resistance to therapy in melanoma

Authors	Samples	Approach	Main Markers and States
Tirosh et al.^17^	-4645 cells (malignant, immune, and stromal cells) from 19 melanoma patients -2068 T cells from 15 melanomas	-Sorting/FACS -sc-RNAseq	-MITF, TYR, PMEL, MLANA (sensitivity to MAPKi) -AXL, NGFR (dormant MAPKi resistant subpopulation) -PD1, TIGIT, TIM3, LAG3, CTLA4 (exhaustion program)
Ho et al.^41^	100 cells from 2 BRAF-mutant melanoma cells -A375 sens vs res -451Lu sens vs res	-sc-RNAseq -FACS/Sorting	-DCT (reduced BRAFi response) -AXL, NRG1 (transition state to BRAFi resistance)
Shaffer et al.^42^	Melanoma cells isolated from 2 patients treated with a BRAFi -WM989 -WM983B	-sc-RNA FISH -Sorting/FACS	-WNT5A, AXL, EGFR, PDGFRB, JUN, NRG1 (transitional state to MAPKi resistance)
Rambow et al.^43^	3 different PDXs from BRAF-mutant melanomas exposed to MAPKi to mimic MRD. -Phase 1 = tumour shrinkage -Phase 2 = impalpable size -Phase 3 = development of resistance	-sc-RNAseq	-MITF, TRPM1, GPR143, MLPH (differentiation/pigmentation state) -SLIT2, BGN, TNC (EMT state) -NGFR, AQP1, GFRA2, RXRG (de-differentiative state) -CD36, SLC7A8, SLC12A7, DLX5 (nutrient-deprived state)
Su et al.^44^	18 patient-derived BRAF-mutant melanoma cells treated with a BRAFi for: -3 days -3 weeks	-proteomic SCBC barcode chip -FACS	-MART1, MITF (pigmented state 3 days after BRAFi exposure) -NGFR (slow-cycling neural crest-like state 3 weeks after BRAFi) -p-ERK, p-NFκB (BRAFi tolerance state 6 days after treatment)
Lun et al.^45^	-Human embryonic kidney HEK293T cells transfected with 649 kinases/phosphatases -A375 melanoma cell lines treated with MAPKi	-Mass cytometry (CyTOF)	-ABL1, BLK, FES, MAP3K2, MAP3K8, MOS, NTRK2, SRC, YES1 (induction of MAPK signalling) -MAP3K8, MOS (resistance to BRAFi) -SRC (resistance to BRAFi/MEKi)
Krieg et al.^46^	40 matched PBMCs derived from a cohort of 20 melanoma patients before and 12 weeks after anti-PD1	-Mass cytometry (CyTOF) -FACS/Sorting	-HLA-DR, CTLA-4, CD56 and CD45RO, CD3, CD27, CD28 (T cell differentiation and activation) -PD-1, IL-4, IFN-γ, IL-10, IL-17A, Grz-B (T cell function) -CD14 + CD16−HLA-DR (myeloid cell function)
Nirschl et al.^47^	333 individual dendritic cells and monocytes sorted from a single lymph nodes of melanoma metastasis	-sc-RNAseq -FACS/Sorting	-IFN-γ, SOCS2 (adaptive anti-tumoral immunity and T cell priming)
Jerby-Arnon et al.^48^	Malignant and T cells: -2.987 from 17 newly collected melanomas, -4.199 from 16 patients previously collected (Tirosh et al)	-sc-RNAseq	-B2M, CTSB, HLA-A/B/C, TAPBP (antigen processing and presentation) -CD47, CD58 (immune modulation) -CD59, C4A (response to the complement system)
Sade-Feldman et al.^49^	16.291 immune cells from 32 melanoma patients at baseline and longitudinally during -anti-PD-1 -anti-CTL4 -combo-therapies	-sc-RNAseq -FACS/Sorting	-TCF7, TIM3 (T cell differentiation, self renewal and memory) -BATF, PRDM1, TOX, HMGB2, IRF2 (CD8+ T cell exhaustion)
Gide et al.^51^	T cells derived from 120 melanoma patients’ biopsies -63 anti-PD-1 monotherapy -57 combined anti-PD1 with anti-CTLA-4	-Mass cytometry (CyTOF)	-CD45RO + EOMES+, TBET (T cell differentiation and memory)

## Conclusions

Tumours are complex ecosystems governed by specific spatiotemporal rules and by interactions between the different cellular components where its diversity represents a source of therapeutic opportunities and failures^53^. The first are best exemplified by the striking clinical success in malignant melanoma of targeted and immuno-therapies^32^. The second is represented by the development of drug resistance, which is fueled by intratumor hererogeneity. Therefore, expanding our knowledge on this phenomenon is crucial for the development of more effective therapeutic approaches and for a better prediction of patient outcomes. We do hope we have provided evidence that the best approach to studying intratumor heterogeneity is by sc analyses. Thereby, it is possible to quantify genetic and transcriptional features present in hundreds to thousands of individual cancer and non cancer cells per tumour.

Briefly, two main points have emerged from sc studies: (1) cancer/stromal cell interactions are informative of MAPKi resistance whereas (2) cancer/immune cell interactions are informative of resistance to immunotherapy. These two resistant conditions share some common transcriptomic events, for instance the so-called IPRES signature^26^. Interestingly, a common denominator of all these studies was understanding the existence of ab initio resistance programmes. This was the case of dormant drug resistant subpopulation characterized by AXL-dependent signatures, which apparently represent the major drivers of the evolution of resistance to MAPKi^17^. These cells mirror the quiescent tolerant cells responsible for the so-called MRD^43^. Also for immunotherapy, a drug resistance program exists prior to starting treatments and is enriched following immune checkpoint blockades in resistant melanomas^49^. This state is associated with T cell exclusion and immune evasion, and distinguishes cold niches, allowing to predict clinical responses to ICIs^48^.

**Fig. 2:**
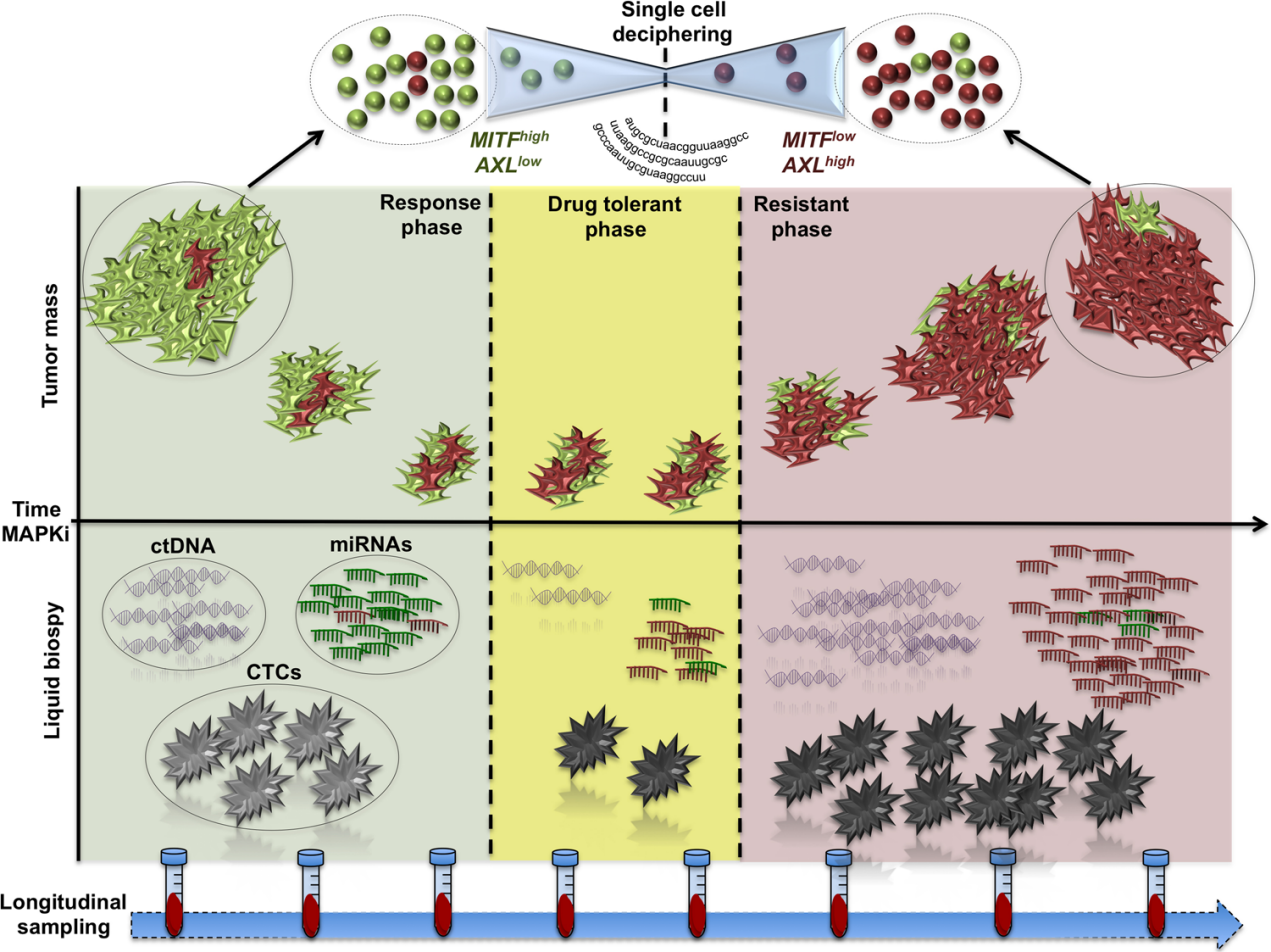
Schematic diagram illustrating single cell analysis implementation with liquid biopsies to gain diagnostic purposes. Bulk melanomas contain a little percentage of dormant drug resistant cells before starting MAPKi treatments, which emerge as a resistant population passing in a drug-tolerant phase during the course of the therapy. Non invasive liquid biopsies may help to longitudinally measure the evolution of the therapy in order to predict the emergence of drug resistance and potentially predispose new tools to counteract it

Despite the power of sc analysis on tumour biopsies, its major limitation is the difficulty in implementing it on a routine basis for diagnostic purposes. Other less invasive approaches are required in order to study tumour heterogeneity and in particular to follow it during disease evolution and after therapy(ies). In the majority of cases, clinical conditions and surgically unaccessible metastatic tumours strongly limit the possibility to obtain tissue re-biopsies to support sc studies. Also, sc studies of tumour biopsies and re-biopsies rely on sophisticated technologies available only in specialized laboratories. To tackle these issues, non invasive longitudinal liquid biopsies derived from patient blood samples are emerging as fundamental tools in solving the shortcomings of tissue sampling^53,54^ (Fig. 2). Among them, the most reliable one is the circulating tumour DNA (ctDNA), which allows to identify tumour specific genetic alterations^55–57^. The ctDNA evaluation accounts for a high specificity/sensitivity with detection rates comparable to those of tissue biopsies. In addition, also non coding RNAs are emerging as valuable tools in effectively diagnosing cancer and responding to therapy^58^. Most of the study candidates have microRNAs whose measure and extraction in human fluids is easy and have been the focus of hundreds of pubblications^59,60^. However, it is important to highlight that the validation of miRNA-based liquid biopsies is limited by several factors such as data normalization and difficulty to interpret^58,61^. Finally, we only mention that microRNAs have also been described as valuable therapeutic tools in cancer management^58,59,62^. The last frontier of the liquid biopsies are circulating tumour cells (CTCs) which can be exploited for (1) transcriptional analysis and (2) integration of ctDNA informations^63^. Indeed, transcriptomic profiles of melanoma CTCs revealed high heterogeneity in gene expression patterns compared to primary tumours valuable to develop novel candidate biomarkers^64^. Sc analysis of CTCs together with that of circulating immune cells, will certainly help to address the evolution of the immune response within the tumour microenvironment^65^.

**Fig. 3:**
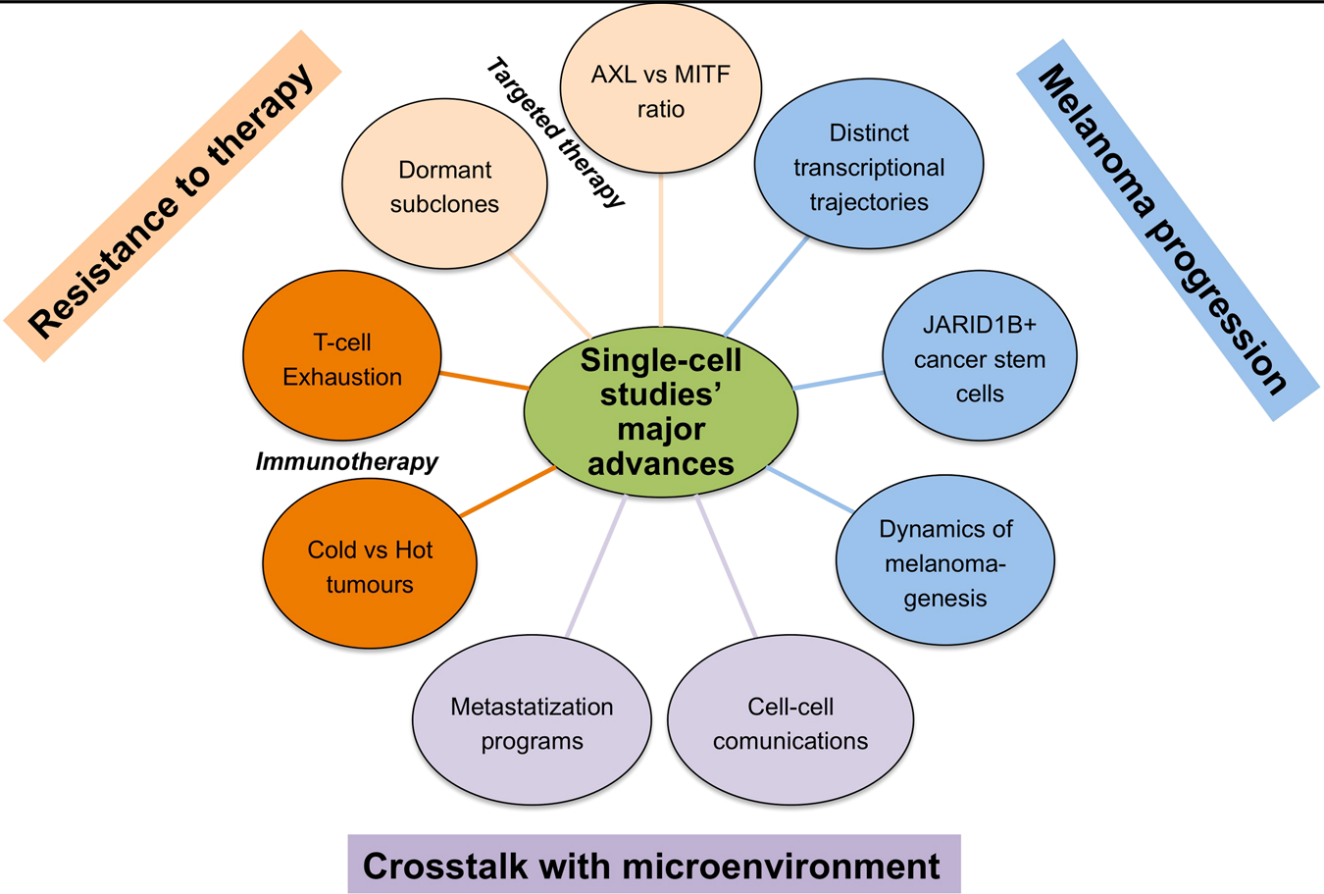
Schematic representation illustrating the major advantages brought by single cell studies to melanoma research. Single cell studies allow to learn several lessons about intratumour heterogeneity which drives melanoma progression as well as the impact of targeted/immuno-therapies through the characterization of the crosstalks with the cellular and non cellular elements of the tumour microenvironment

In summary, using metastatic melanoma as prototypical example, we believe we provided enough evidence to sustain the concept that the era of precision medicine will be dominated by sc analysis at several levels (Fig. 3). This knowledge and translation into the clinic is expected to boost patient response to emerging therapies and allow the development of powerful combinations and at the same time avoid unnecessary treatments for intrinsically resistant tumours.
